# Payer reimbursement practices and incentives for improving interpretation of germline genetic testing

**DOI:** 10.1093/jlb/lsad020

**Published:** 2023-07-09

**Authors:** Patricia Deverka, Janis Geary, Charles Mathews, Matan Cohen, Gillian Hooker, Mary Majumder, Zuzana Skvarkova, Robert Cook-Deegan

**Affiliations:** Deverka Consulting LLC, Chapel Hill, NC, USA; Consortium for Science, Policy & Outcomes, Arizona State University, Washington, DC, USA; Clearview Healthcare Partners, Newton, MA, USA; Clearview Healthcare Partners, Newton, MA, USA; Concert Genetics, Nashville, TN, USA; Baylor College of Medicine, Houston, TX, USA; Consortium for Science, Policy & Outcomes, Arizona State University, Washington, DC, USA; Consortium for Science, Policy & Outcomes, Arizona State University, Washington, DC, USA

**Keywords:** cancer, genetic testing, coverage, reimbursement, data sharing, precision medicine

## Abstract

Germline genetic testing for inherited cancer risk has shifted to multi-gene panel tests (MGPTs). While MGPTs detect more pathogenic variants, they also detect more variants of uncertain significance (VUSs) that increase the possibility of harms such as unnecessary surgery. Data sharing by laboratories is critical to addressing the VUS problem. However, barriers to sharing and an absence of incentives have limited laboratory contributions to the ClinVar database. Payers can play a crucial role in the expansion of knowledge and effectiveness of genetic testing. Current policies affecting MGPT reimbursement are complex and create perverse incentives. Trends in utilization and coverage for private payers and Medicare illustrate opportunities and challenges for data sharing to close knowledge gaps and improve clinical utility. Policy options include making data sharing (i) a condition of payment, and (ii) a metric of laboratory quality in payment contracts, yielding preferred coverage or enhanced reimbursement. Mandating data sharing sufficient to verify interpretations and resolve discordance among labs under Medicare and federal health programs is an option for the US Congress. Such policies can reduce the current waste of valuable data needed for precision oncology and improved patient outcomes, enabling a learning health system.

## I. INTRODUCTION

Germline genetic testing is recommended in practice guidelines^1^ to identify individuals who can benefit from targeted risk-reducing interventions or to select an appropriate therapy in patients already diagnosed with cancer.^2^ Whereas in the past, germline testing focused on specific genes such as *BRCA1* or *BRCA2* to evaluate risk of breast, ovarian, and other cancers, over the past decade, there has been a shift to using multi-gene cancer panel tests that can reveal inherited risk for one or more cancer types. Several large laboratory-based studies have demonstrated that there is substantial genotypic heterogeneity among individuals referred for germline genetic testing, such that many pathogenic variants would be missed if testing were limited to specific genes such as *BRCA1/2* alone.^3^

The absolute number of genes included as part of multi-gene panel tests (MGPTs) has also increased over time. Two factors account for this trend. First, there is greater reliance on next-generation sequencing (NGS) platforms as the cost of sequencing has dropped a millionfold over the past decade, making testing for multiple genes more efficient than one- or two-gene testing. Second, studies have indicated that mutations in an expanding number of genes are associated with increased cancer risk, providing clinical justification for testing multiple genes to avoid missing clinically actionable variants. This movement is continuing, with emerging evidence supporting the use of all-exome sequencing^4^ and whole genome sequencing (WGS),^5^ depending on the population targeted for testing.

Most hereditary cancer testing is now conducted using MGPTs that report on variable numbers of genes depending on the particular test ordered.^6^ While a recent study noted nearly 300 cancer-related MGPTs currently on the market from 17 laboratories, these tests varied widely in the number and choice of genes included, with one MGPT covering up to 607 genes.^7^ The most frequent clinical applications are for inherited forms of breast or ovarian cancer and colorectal cancer, as well as general cancer testing. Although there are resources such as the gene-disease validity database from ClinGen^8^ and practice guidelines,^9^ there are no standard criteria for directing the addition of more genes to MGPTs, as demonstrated by the observed heterogeneity in panel composition. There are also uncertainties regarding whether to focus MGPTs narrowly on a specific clinical presentation, such as breast and ovarian cancer risk, or more broadly on overall inherited cancer risk. Finally, standards are lacking for deleting genes from panels as evidence continues to evolve and demonstrates the lack of clinical relevance.

While larger MGPTs detect more pathogenic variants (PVs) that indicate cancer risk, they also detect more variants of uncertain significance (VUSs) that complicate the interpretation of test results.^10^ Notably, detection of VUSs often outnumbers detection of known PVs, with a meta-analysis of breast cancer risk patients demonstrating a ratio of VUSs to PVs of 2.5.^11^ In the long term, such VUS findings play an important role in furthering our understanding of the genetic causes of cancer, but in the near term, VUS findings muddy clinical decision-making and may also introduce consequences that include patient harms: anxiety, unnecessary clinical interventions, and associated adverse events. These have downstream implications for health system costs.^12^ A recent review of the clinical consequences of VUSs indicates that these results not only do not resolve the clinical question that prompted testing but also that only a small fraction (<10 per cent) resolved over a 10-year period.

The inability to determine the pathogenicity of VUSs is a serious limitation to improving the positive predictive value of MGPTs. No single laboratory can aggregate sufficient evidence over time from diverse patient groups to improve interpretation. To standardize variant interpretation and address the problem of VUSs, the National Institutes of Health funded ClinVar to support community sharing of variant data and classification methods.^13^ Evidence-based variant interpretation is resource intensive, requires standardized approaches, and improves with access to the ClinVar database, as well as interpretive consortia including ClinGen, ENIGMA, and InSIGHT.^14^

What is the evidence that sharing data can improve the effectiveness of genetic testing for inherited cancer risk? Casaletto, Cline, and Shirts modeled reclassification of VUSs with and without sharing data.^15^ Reporting a rare *BRCA1*/2 variant along with its risk classification—the information provided to ClinVar—dramatically increases the ability to classify. Linking to case-level clinical data further improves that, a prospect enabled by resources such as ClinVar and BRCA Exchange that include pointers to where case-level data might be found. Sharing rare (one in 100,000) variants boosted classification from 25 to 80 per cent in 1 year and nearly all variants within 5 years in their model. Variants occurring one in a million are harder to classify, but classification is hopeless if it relies on an individual laboratory’s dataset. Sharing becomes even more important when considering that the number of variants predicted but still waiting to be discovered in actual patients range from 45 per cent in the most-studied populations to 88 per cent in others; that is, most variants have not yet been revealed through clinical or research testing,^16^ despite *BRCA1/2* being among the most studied genes in the human genome.

Despite the known clinical benefits of sharing data, laboratories cite numerous hurdles to public data sharing, such as a lack of staff and time, leading to unrecouped costs and other financial burdens.^17^ These barriers could be at least partially addressed if there were additional incentives from stakeholders, especially payers, for laboratories to share both test results and the evidence base for interpretation.^18^ The rationale is that payers are already reimbursing for tests that are not optimized for clinical interpretation, creating the potential for clinical and economic harms from a growing number of VUSs. That is, information is being generated in genetic testing that is not available to build knowledge over time and improve interpretation; this is an avoidable waste of valuable information. This point is demonstrated in Cook-Deegan et al., in this issue, which showed that of 33 companies that have MGPT for hereditary breast and ovarian cancer (HBOC) or Lynch syndrome, 27 per cent share no data with ClinVar, and an additional 51 per cent have not shared variants of at least one gene included on their MGPTs.^19^

Payers could play a key role in improving VUS interpretation by ensuring information does not go to waste. Increased data sharing could foster a virtuous cycle where more data would foster more informative MGPTs, leading to greater competition in marketed tests, which in the end could represent a benefit for payers focused on value-based pricing. Today, current laboratory practice relies on soft ethical norms to promote data sharing in a voluntary system.

One strategy to encourage greater data sharing is for payers to only reimburse for tests performed by laboratories that agree to share data.^20^ Aetna, for example, requires laboratories in their network to deposit genetic test data into ClinVar^21^ in support of improving test quality over time. To support efforts like this, ClinGen also makes available a list of clinical laboratories that meet the minimum requirements for data sharing to support quality assurance.^22^ Underscoring the clinical and economic benefits of greater data sharing for MGPTs that are already reimbursed by payers is the main justification for our policy arguments, in addition to the obvious ethical advantages of advancing collective benefit by creating a robust knowledge commons.^23^

The purpose of this paper is to first illustrate the complexity of reimbursement for MGPTs by describing the framework used by payers for coverage determinations and the coding process for payment. Next, we describe trends in germline cancer genetic testing utilization and coverage for both private payers and Medicare that illustrate the opportunities and challenges for increased data sharing to close the data gaps that could improve clinical utility over time. Finally, we assess policy options that both encourage laboratories to contribute deidentified data to build a robust genetic testing knowledge commons to improve interpretation of VUSs while also using this real-world data to optimize decision-making for both clinical care and coverage determinations. The benefit to all stakeholders is improved interpretation of variants associated with inherited risk of cancer and stronger evidence to guide utilization of MGPTs to improve patient health outcomes. While outside of the focus of this paper, these policy options could apply to other non-cancer forms of testing.

## II. PAYER ASSESSMENT AND REIMBURSEMENT OF MGPTs

The process for obtaining reimbursement for an MGPT involves three interrelated components: coverage, coding, and payment (reimbursement). When determining whether to cover a new MGPT, payers use the same criteria that are used to determine the medical necessity of any test. Specifically, payers review published studies and technology assessments that describe the analytic validity, clinical validity, and clinical utility of the test.^24^ Analytic validity refers to whether the test is accurate and reliable; it reflects the technical performance characteristics of the test. Clinical validity describes whether the test result is medically meaningful and includes concepts such as sensitivity (whether it is positive when it should be), specificity (whether it is negative when it should be), and positive and negative predictive values (whether it detects or rejects what it should, and only that). Clinical utility is dependent on demonstrated analytic and clinical validity but goes further to include evidence that results from the test affect clinical decision-making and improve patient health outcomes.^25^ Studies to evaluate clinical utility are inherently comparative (either comparing MGPT to another genetic test or to a ‘no testing’ strategy) and require measuring both the benefits and harms of MGPTs. Comparative effectiveness studies are rarely conducted in genetics, and lack of clinical utility data is one of the most common reasons that payers cite as justification for their decision not to cover MGPTs.^26^

Payers also rely on testing recommendations included in clinical practice guidelines, as well as recommendations from the US Preventive Services Task Force (USPSTF) that focus on cancer risk mitigation. A positive USPSTF recommendation (A or B grade) means that private payers must provide coverage to their members who meet criteria for testing and is a pathway for Medicare coverage assuming that testing fits one of the existing legislative exceptions for screening tests. Currently, only *BRCA1/2* testing (not more comprehensive breast cancer MGPTs) is recommended for use in high-risk women by the USPSTF.^27^

Additional payer considerations include whether a test is recommended in guidelines developed by professional societies such as the National Comprehensive Cancer Network, the American Society for Clinical Oncology, and the American College of Obstetrics and Gynecology. Other influential sources of evidence reviews for payers include health technology assessments conducted by organizations such as the ECRI Institute^28^ and Hayes^29^ that provide subscription-based access to their reports. Since MGPTs and other complex genetic tests such as whole exome or genome sequencing require specialized genetics expertise, payers may also partner with a laboratory benefit manager (LBM) who has focused utilization management programs for these tests. Payers can use LBM evidence-based coverage policies in whole or in part to make decisions about coverage and reimbursement. A potential downside of the LBM business model is that these organizations are often compensated based on reducing ‘inappropriate’ genetic testing utilization, creating a financial interest in denying services without commensurate analyses of the impact on patient health outcomes.^30^

Importantly, MGPTs present specific coverage challenges to payers because when additional genes are included in the test, the relationship between test results and impact on care is less well understood. This is because there is a lack of clinical utility evidence for some genes, while there is stronger evidence for other genes. For example, only 16 per cent of the genes included in MGPTs for hereditary cancer were also found on any clinical guidelines or had a gene–disease relationship assessment confirmed via ClinGen.^31^ In addition, there are interpretation and management challenges regarding VUSs. As noted in a companion paper in this special issue, the current understanding of potential risk mutations is highly Eurocentric, lacking data on underrepresented groups that exacerbates the VUS problem in non-European ancestry populations.^32^ For payers that cover diverse populations, as is the case for most payers in the USA, these evidence gaps further undercut the potential clinical utility of MGPTs. Thus, for several reasons, MGPTs do not fit payers’ coverage frameworks that must conclude in a binary decision of either medically necessary and covered or investigational/experimental and not covered. The result is that frequently, payers do not cover MGPTs, citing lack of evidence of clinical utility. Nevertheless, MGPTs are frequently ordered by clinicians and reimbursed by payers as described below.

Concerning coding, laboratories must identify a Current Procedural Terminology (CPT®^33^) code that allows them to bill for a test and ultimately obtain payment.^34^ For MGPTs, coding is complicated. Prior to 2013, there were not-specific codes for this category of tests given the rapid evolution of molecular diagnostic testing. Instead, laboratories would use individual codes to bill for each step in the technical testing process included in a test, a practice known as ‘code-stacking’ that is problematic as it frequently leads to overpayment by payers. Until 2013, for example, a *BRCA1/*2 test entailed billing according to the number of ‘amplicons’, or DNA segments sequenced in the process. Myriad Genetics, the only laboratory openly doing commercial *BRCA1/2* testing in the USA until 2013, billed for more than 80 amplicons, and tests for the several genes associated with colorectal cancer risk were similarly billed using code-stacking by Myriad and other laboratories.^35^

Over the last decade, analyte or entity-specific codes have been developed (often requiring years of work to secure) that can be used to bill for one or a few gene tests (eg *BRCA1* and *BRCA2*), or for MGPTs (eg GSP or genome sequencing procedure codes). A test from a particular laboratory can be given a PLA or proprietary laboratory analysis code.^36^ However, many laboratories still use stacks of single-gene codes and generic codes when submitting claims for payment, rather than using codes for the panels, thereby undermining the intended effects of greater transparency and specificity in coding practices. One reason that code stacking is still frequently used is that the payment rates are frequently much higher and more likely to be covered than a specific MGPT code, and influential payers such as Palmetto (a southeast regional Medicare Administrative Contractor) also incentivize the use of a generic code that can be mapped to a separate coding system called DEX-Z codes^37^ to facilitate payment. For example, Medicare currently pays $1117.98 for a hereditary breast cancer panel (CPT code 81432) while reimbursing $1824.88 for *BRCA1/2* gene analyses alone (CPT code 81162), which might be combined with $584.23 under code 81164, a test for structural *BRCA1/2* rearrangements.^38^ This creates a perverse incentive for laboratories to use less accurate coding in order to maximize payment or to do tests in ways that generate more revenue but less information. It prices tests that produce more information—and results that could inform future interpretation—lower than less informative two-gene testing. The current system wastes the information that is generated but not reported on genes increasingly tested for inherited cancer risk, constricting the flow of information needed to reduce the number of VUSs and improve interpretation over time. Current practice thus impoverishes the knowledge commons.

The persistent use of unlisted molecular pathology procedure codes as well as general codes for rarer diseases (Molecular Pathology Tier 2 CPT codes)^39^ frustrates efforts to create a transparent and predictable testing-and-reimbursement framework. While many payers do not officially ‘cover’ MGPTs to assess cancer risk, according to their published medical policies,^40^ they often do actually pay for these tests because they lack the tools to accurately identify them based on claims submitted by laboratories. The lack of coding transparency for the multitude of MGPTs currently available to clinicians is a key reason that payers have difficulty tracking what tests they are actually paying for.

Finally, once a new code becomes available, Medicare has a formal structured and semipublic process for payment assignment, a reimbursement schedule.^41^ Private payers may consider the Medicare rate when developing their own payment approach but typically employ both cost-based and value-based strategies. The cost-based approaches include (i) a comparison of the test content to existing tests with established payment rates and (ii) a rough or detailed crosswalk to tests that use similar methodologies or (iii) an actuarial approach that takes into consideration how many patients are expected to receive the test. The value-based approach is also known as the health economics approach because decision-makers weigh the test cost relative to the impact on health outcomes.

The Centers for Medicare and Medicaid Services (CMS) establishes payment for new CPTs, using several different procedures that depend on whether the test considered a Clinical Diagnostic Laboratory Test (CDLT) or an Advanced Diagnostic Laboratory Test (ADLT). MGPTs do not meet criteria for ADLTs because this designation specifically refers to tests that analyze DNA, RNA, or proteins combined with a unique algorithm to yield a patient-specific result. For CDLTs, CMS uses one of two methods: (i) crosswalking when a new test is determined to be similar to an existing test or (ii) gap-filling when no comparable test is available.^42^ Payment codes are then adjusted, based on private payers’ rates under PAMA (Protecting Access to Medicare Act) [P.L. 113-93, §216].^43^ Since the passage of PAMA, Medicare’s previous local fee schedules for lab tests have been replaced with a single national fee schedule.^44^ Starting in 2018, the amount paid by Medicare for most tests is equivalent to the weighted median of private payer rates, and these amounts are revisited every 3 years. PAMA also includes a phase-in of payment reductions starting in 2022 that was delayed until 2023.^45^ PAMA represents a major shift in the payment framework for diagnostics in which the Medicare reimbursement rate is benchmarked to commercial payer rates. In sum, the process for obtaining payment for MGPTs from private payers and Medicare is highly complex. Also, many steps in the current process are not built to scale with the pace of technological advancement. Coverage depends on payer review of practice guidelines and the medical literature; payment depends on coding and fee schedules that are periodically adjusted.

## III. OVERVIEW OF GERMLINE CANCER GENETIC TESTING UTILIZATION

We used empirical data to gain insight into how companies bill for HBOC and Lynch syndrome MGPTs. The detailed methods, data, and results can be found in Supplemental Methods.^46^ We analyzed CMS payment^47^ and costing^48^ data for molecular pathology and a list of CPT codes associated with HBOC/Lynch syndrome panels extracted by Geary et al.^49^

Eleven of the companies included in the Geary et al. paper had CPT coding data available (see Table 1).^50^ The average number of unique CPT codes each company used for its panels ranged from 1 to 12, indicating substantial variation in how these tests were billed. While all of the HBOC tests we included were panels (ie included more than just *BRCA1/2* testing), only three companies billed using the 81432 code ($679) for breast cancer panels and five used the more expensive 81162 *BRCA1/2* code ($1825).^51^ Our estimates are imprecise because some tests use the undefined code 81479, but the estimated average amount billed by each laboratory for panels ranged from $679 to $8589.

**Table 1:** Characteristics of HBOC and Lynch syndrome MGTPs offered by 11 companies with available CPT code data

	# genes included on HBOC/Lynch MGPTs	# HBOC/Lynch Variants shared	# genes with no variants shared	Average # unique codes	Average # total codes	Average cost of HBOC MGPTs^*^	Average cost of Lynch syndrome MGPTs	# panels using CPT Code 81162	# panels using CPT Code 81432	# panels using CPT code 81479
Prevention Genetics	14	996	0	11.9	28.0	8589.17	3417.85	6	0	7
Quest Diagnostics	16	5455	1	9.0	9.0	n/a^**^	3417.85	0	0	0
LabCorp	25	7917	1	7.0	7.7	5808.23	2037.20	3	1	3
Ambry Genetics	26	37,067	1	7.4	7.4	4691.54	3417.85	4	0	0
GeneDx	25	21,487	0	4.3	4.3	2806.07	3029.15	3	0	0
Mayo Clinic Laboratories	23	778	11	4.0	4.0	Uses CPT 0102Ux, no cost associated	3664.15	0	0	0
University of Washington Medical Center	81	1118	33	3.0	3.0	2994.68	n/a	1	0	0
University of Chicago Genetic Services	20	1457	0	2.8	2.8	898.36	3232.65	0	0	0
Fulgent Genetics	25	726	0	2.7	2.7	2137.41	n/a	0	0	0
ARUP Laboratories	24	1666	1	2.0	2.0	1117.98	n/a	0	1	0
Blueprint Genetics	28	19	25	1.0	1.0	679.05	n/a	0	1	1

^*^Calculated using CMS billing and therefore does not include costs for the 81479 code as there is no billing amount associated with that code.

^**^N/A indicates the company did not have a panel that fit the criteria (ie did not have a Lynch syndrome panel or an HBOC panel with more than two genes).

Partial information about payer expenditures associated with these tests was revealed by examining the number of services and payments billed to CMS (Medicare) associated with frequently used CPT codes. We compared the amount of money paid through CMS for the most-often-used CPT codes in our database for both HBOC and Lynch syndrome tests, as well as the undefined 81479 code. Payment of the undefined code soared from approximately $4 million in 2013 to $288 million in 2020. Billing for several CPT codes associated with Lynch syndrome (there is no code comparable to the HBOC codes) also increased substantially in 2018–19, although they dropped sharply in 2020, presumably related to pandemic-related reduction of cancer screening in general. Expenditures under the more expensive *BRCA1/2* non-panel CPT code 81162 grew consistently until peaking at $118 million in 2019, costing over 10 times more than the amount paid that year for the 81432 breast cancer panel code. This occurred even though labs had in practice largely shifted to multi-gene NGS, replacing PCR-based sequencing of just *BRCA1/2* starting in 2012. That is, coding and reimbursement do not map cleanly to the procedures labs are actually using to do their testing.

Using publicly available data sources, it remains unclear how often each of these companies is conducting these tests and billing Medicare and private insurers for them, exactly how much they are getting reimbursed for, and how many data on how many genes are being produced. However, it is clear that despite the increasing billing over time and high prices requested for these tests, the data that are produced from these tests are often not made available through resources like ClinVar. As described in the overview paper, the majority of companies that offer HBOC and Lynch syndrome tests are either not sharing any data to ClinVar, or not sharing data on at least some of the genes included on their tests. Given the fact that in every submission to ClinVar there are new, never- reported BRCA 1/2 variants (eg 2755 BRCA1 and 5288 BRCA2 variants added to ClinVar between May 2020 and May 2023), knowledge about even these two heavily studied genes is far from complete.

## IV. COMMERCIAL AND MEDICARE COVERAGE OF GERMLINE GENETIC TESTING AND MGPTs

### IV.A. Private Payers

To better understand the payer coverage landscape for germline testing, including testing for one or a few genes (eg *BRCA1* and *BRCA2* for HBOC) and MGPTs, we completed a review of coverage policies of the 15 largest payers (in terms of covered lives) and examined Medicare national coverage determinations and local coverage determinations (see Table 2). We observed that *BRCA1/2* testing is almost universally covered for those with a personal or family history in a first-degree relative (eg personal cancer diagnosed before age 50, family history of breast cancer, recurrent cancer, or those with ovarian cancer). Breast and ovarian MGPTs (referred to as HBOC panels) are not covered as widely, and when allowed, there are often numerous conditions for coverage. HBOC panel coverage conditions included (i) similar to *BRCA1/2* testing, (ii) coverage if certain genes are included (eg if *BRCA1* and *BRCA2* are included), and (iii) coverage of only the *BRCA1/2* portion of the panels. Of the 15 policies examined, 6 allow for testing with *BRCA1/2* two-gene requirements, whereas five payers will only cover the BRCA portion of a panel. For example, Cigna specifically alludes to expanded coverage of panel testing when favorable contracting arrangements have been made. This then allows for coverage of the full panel. Cigna will also cover some MGPTs when the posted medical necessity criteria for the panel are met for at least one or a few genes on the panel (eg *BRCA1/2*).^52^ Florida Blue Cross Blue Shield was the only payer that does not cover HBOC panels in any capacity. Taken together, these findings point to broad support of two-gene testing of HBOC and extensive hesitancy to provide explicit coverage of broader HBOC panels.

**Table 2:** Coverage determinations

Plan name	# of covered lives (2022)^74^	Coverage of BRCA 1/2	Coverage of HBOC MGPT	Coverage of Lynch	Coverage of pan-tumor MGPT
UnitedHealthcare	26,580,000	Covered with family or personal history	Covered with family or personal history	Covered with family or personal history	Covered with family or personal history
Anthem	16,694,190	Covered with family or personal history	Cover only BRCA portion	Covered with family or personal history	Not covered
Aetna	16,638,652	Yes	No	Yes	Yes, select panels
Cigna Corporation	13,853,996	Covered with family or personal history	Covered with family or personal history	Covered with family or personal history	Not covered
Kaiser Permanente	9,440,374	Not posted			
BCBS Illinois	7,590,694	Covered with family or personal history	Covers only BRCA portion	Covered with history of cancer	Not covered
Blue Cross Blue Shield Texas	5,828,267	Covered with family or personal history	Covers only BRCA portion	Covered with history of cancer	Not covered
Blue Cross Blue Shield Michigan	5,241,681	Covered with family or personal history	Not described	Covered with family or personal history	Not described
Florida Blue (Blue Cross and Blue Shield of Florida, Inc.)	3,147,518	Covered with family or personal history	Not covered, considered investigational	Covered with family or personal history	Not covered
Blue Shield of California	2,923,785	Covered with family or personal history	Covered with family or personal history	Covered with family or personal history	Not covered
Highmark Pennsylvania	2,717,969	Covered with family or personal history	Covers only BRCA portion	Covered with family or personal history; no coverage for portion of test previously done	Not covered
Centene Corporation	2,602,601	Covered with family or personal history	Covered if BRCA included	Covered with family or personal history	Not covered
CareFirst Blue Cross Blue Shield	2,473,798	Covered with family or personal history	Not described	Covered with family or personal history	Not covered
Horizon Blue Cross Blue Shield New Jersey	2,454,773	Covered with family or personal history	Covered with family or personal history	Covered with family or personal history	Not covered

^*^Source: Policyreporter, quarterly filing of health plans. https://www.policyreporter.com/payer-data/ (accessed 15 June 2023).

Often, the policies have vague language about what is considered eligible for a case-by-case review of broader genetic testing, rather than outright support for panel-based testing. Coverage policies also vary in which genes are included, an observation confirmed by other researchers studying payer coverage of MGPTs for hereditary cancer genes.^53^ Some commercial plans cover select genes but not others. For example, Anthem’s policy covers a subset of the genes on Invitae’s Breast and Gyn Guidelines-Based Panel (eg *BARD1* and *PTEN*) but not others (eg *MLH1* only for Lynch syndrome). This presents challenges for labs that hope to offer comprehensive cancer risk assessment and treatment guidance.^54^

For other colorectal cancers, Lynch syndrome panels are universally covered for individuals with early onset (<age 50) colorectal or endometrial cancer or family history (a first- or second-degree relative with confirmed HNPCC mutation) as defined by guidelines (Amsterdam II or Bethesda). Some plans state they will only cover genes that have not been tested previously by the same methodology.

Broader panels marketed as simply for ‘hereditary cancer risk’ (ie not-specific cancer syndromes) do not appear to have broad coverage outside of select conditions for predisposition to cancer risk (eg Li–Fraumeni syndrome).^55^ Importantly, of the 15 plans analyzed, only UnitedHealthcare covered pan-tumor screening (indicated by CPT code 0134U), with many plans citing this test as investigational and not medically necessary, likely due to questions about the clinical utility of this type of testing outside of HBOC and Lynch.

Enforcement of payer policies is limited by what can be identified via CPT coding. As described above, payers face difficulty in assessing which tests qualify for coverage because CPT codes only capture certain types of information (eg completion of a panel) but not which genes are included. In most cases, the policies simply list the *BRCA1/2* and Lynch-gene CPT codes (eg 81435 and 81436) as explicitly covered. Then, other tests using different or additional codes would need to be reviewed for coverage. Some labs have secured unique proprietary analytic codes (PLAs) to describe their specific offerings, which facilitates test-level coverage determinations [eg 0238 U, which describes Variantyx’s PCR-free WGS platform with algorithmic data analysis to evaluate the five genes listed in the code for variants relevant to Lynch syndrome].

### IV.B. Medicare

Medicare’s coverage for this category of testing is bound by Medicare’s statutory constraints. The original statute supported only reimbursement for medically necessary care for patients with current illness. Individual screening services have been added to the program over time through amendments to the statute (eg colonoscopy and PSA). Therefore, germline testing conducted for the purposes of screening in the asymptomatic population is excluded. However, Medicare will cover *BRCA1* and *BRCA2* testing for individuals with signs and symptoms of breast, ovarian, or other hereditary cancer syndromes once they have been diagnosed or if they have been previously diagnosed. There are now Medicare coverage pathways for patients with a personal history of either breast or ovarian cancer (BRCA) testing, and Lynch syndrome testing is covered in some regions.^56^

Similarly, multi-gene panels can be covered by Medicare for patients diagnosed with cancer if they are aligned to the indications and limitations of coverage listed in the National Coverage Determination (NCD) created to provide coverage of comprehensive genomic profiling (§90.2 NGS). This policy will also apply to genetic testing for susceptibility to breast or ovarian cancer when performed and aligned to the NCD. Given this is a national coverage determination, it will have an important influence on coverage of multi-gene panels for Medicare beneficiaries that have chosen to enroll in the Medicare Advantage program (approximately 42 per cent of beneficiaries). In the summer of 2022, Palmetto/MolDX published a local coverage determination (LCD) addressing hereditary cancer panel testing broadly, replacing LCDs for *BRCA1/2* and Lynch syndrome.^57^ The policy is complex and focuses on the requirements for panels to undergo technical assessments and provide specific gene content criteria. It is also very prescriptive in how these tests should be coded. However, there is no overarching Medicare coverage of prognostic germline testing and most Medicare Advantage offerings can decide what types of prognostic tests to cover for these beneficiaries without a superseding national Medicare policy, provided they are not more restrictive than the LCDs or NCDs.

In summary, coverage of advanced hereditary cancer testing is limited by both commercial plans and the Medicare program. Although commercial payers consistently provide coverage of the more-well-established *BRCA1/2* and Lynch syndrome panels, their perspectives on coverage of expanded panels are generally negative with only a few plans supportive of coverage and often, coverage is linked to performance at a preferred lab. Additionally, the Medicare program’s historic lack of focus on prevention and prognosis in otherwise asymptomatic individuals has resulted in non-coverage of hereditary test options in patients without a direct personal history of cancer.

## V. POTENTIAL POLICY SOLUTIONS

As labs respond to their customer’s demands, clinical preference for and utilization of MGPTs continue to grow. The clinical utility is well known for disease-associated variants in well-characterized genes such as *BRCA1/2* but can be compromised by poorly understood VUS in BRCA genes, and the problem is larger in other less-studied genes. Labs seeking to validate their offerings suffer from a lack of data about new genes and a lack of standardized criteria for adding genes to panels. Different labs test different genes, with only their own internal databases and several public open databases to guide interpretation.

Ostensibly, labs are incentivized to only offer tests that have consistent reimbursement but there is a current disconnect between the coverage framework used by payers to determine whether MGPTs are medically necessary and actual reimbursement practices. The reasons for this are multifactorial, including the lack of specific-enough CPT codes, the complexity and quality of genetic assessments, coverage policy gaps as well as implementation challenges, and the shortage of genetics expertise inside payer organizations. Accordingly, currently labs primarily bill for their services using a limited subset of codes that describe only the most well validated components of their panels (eg *BRCA1/2*). Yet the actual tests that are performed may generate much more information than is reported, and a very small fraction of new information flows into public databases to build a knowledge base for future interpretation.

The question is not whether to cover any testing but what should be done with the information generated by the tests that are being paid for today. The additional information from MGPTs comes at a very low marginal cost, as samples are processed for both *BRCA1/2* and other genes at the same time and the laboratory procedures generate far more information than is captured for use in interpretation. We advocate keeping the same reimbursement levels paid today by payers, but developing a mechanism to channel the additional information generated by these tests into public databases where it can be interpreted and improve the system over time. This comports with the 2011 ‘Precision Medicine’ study from the National Academies^58^ and is reinforced by more recent proposals for building a system to integrate multi-parameter molecular data with clinical, environmental, and outcome data to support both patient care and biomedical research.^59^ In parallel, there are formal statements from professional societies about the need for data sharing by clinical laboratories in order to improve genetic health care.^60^^,^^61^ The common theme is a need for policies that support greater data sharing in order to take full advantage of information being generated in the process of care delivery.

We are not advocating for more liberal coverage policies for MGPTs or a world in which ‘anything goes’ in genomic testing. At this time, it is not at all clear how many genes it would make sense to test for purposes of identifying individuals to receive risk-mitigation strategies or targeted cancer therapies, precisely because the necessary knowledge base is still developing and so we do not know what genes or variants are associated with which outcomes. Rather, our argument centers on building the knowledge base (what genes to include on tests and how to interpret variants). If there were consistent public data sharing, it would create a positive feedback loop where improved understanding of how test results impact provider behavior and patient outcomes would inform better interventions over time.

A knowledge commons anchored in public databases such as ClinVar would be an efficient mechanism to fill gaps in evidence. There could also be an opportunity to align the interests and incentives that will benefit multiple stakeholders including patients and laboratories. For example, patients will benefit if the system gets better over time and cancers are avoided or unnecessary surgeries from harmless variants are averted. Also, labs would benefit if data sharing were viewed as an element of systematic policies to improve the quality and utility of laboratory results. Additionally, there could be opportunities for lab-to-lab networking that resolve discrepancies in interpreting results.^62^ If any issues are identified as part of quality control assessments, these could be addressed by reviewing data from the open data resources/repositories within a knowledge commons.

A payer would benefit from leveraging a data commons in several ways. The quality of lab tests would improve by reducing the number of VUS results while ensuring that there are many strong and competitive offerings in the market place to keep costs down. The main policy recommendation for payers is to ‘promote data sharing through contractual and/or condition-of-payment provisions’. An example of the first option (contractual approach) is the creation of preferred laboratory networks^63^ supported by Concert Genetics. Laboratories gain entry into the preferred network by completing a quality-focused questionnaire that includes questions regarding data-sharing practices. Payers then couple narrow, qualified networks in combination with more transparent reimbursement policies to ensure that payers reimburse for high-quality MGPTs with actionable results. In addition to improving MGPT quality, payers will save money over time as labs compete to be part of the preferred network. By building in a quality improvement component, this approach also allows payers to count these efforts as an element of a medical service (as opposed to administrative costs, which are capped), which is financially attractive to payers. The hereditary cancer LCD published by Palmetto/MolDX has also taken steps to address quality in bundling the requirement for technical assessments with coverage and could be a natural place to also create incentives for data sharing given the improved clinical validity that could result.^64^

A second option for payers is to condition coverage or provide higher test reimbursement to laboratories that agree to share MGPT variant data in the knowledge commons. If CMS were to adopt this posture (eg mandate data sharing of uncharacterized variants and of known variants sufficient to verify interpretation for tests reimbursed through Medicare), private payers might follow suit. A secondary benefit of this approach is that the knowledge commons would become a viable source of real-world data so observational studies could be conducted to evaluate the clinical utility of MGPTs. The creation of real-world data would specifically address the concerns of researchers and manufacturers to avoid conducting large randomized controlled trials that may be infeasible due to cost and time constraints. A related approach is to condition coverage on the collection of real-world data on variants that cannot be readily interpreted as part of a program known as coverage with evidence development or CED. CED refers to a situation where a payer provides provisional coverage of certain interventions conditional on further collection of population-level evidence from a prespecified study.^65^ Outside of single payer systems such as Medicare, it is unlikely that private payers would undertake the complexity of administering a study given the restrictions imposed by various plan benefit designs and the large sample sizes needed to demonstrate clinical utility. Moreover, since many MGPTs already are being reimbursed, there are few incentives for laboratories to pursue coverage with evidence development.

An interview study of payers focused on understanding their approach to MGPT coverage determinations revealed that payers recognize that MGPTs are often used in a hybrid research/clinical setting because clinical utility information is unevenly distributed across the genes in the panel.^66^ This leads to a situation where payers are covering tests that include information about less-studied genes in a setting that lacks the rigor of a clinical study. While ideally, all necessary clinical utility studies would have been conducted prior to the clinical availability of MGPTs, payers understand that appropriately designed studies may require thousands of patients followed for several decades. Therefore, although payers had concerns about covering MGPTs in a manner that blends clinical care and evidence generation (ie research), a sizable number were open to supporting use of hereditary cancer panels that combined more- and less-studied genes within a model that fostered transparency to enable rigorous research. For example, there could be grants from payer company foundations or collaborations between large self-insured employers and payers to underwrite a research study.^67^ One important caveat is that some payers worried about cases where more definitive evidence down the road would create uncertainties regarding how to recontact these patients. Requirements for data sharing and consistent standards for informed consent and recontact could be part of such a model and would bolster transparency.

A similar qualitative interview study^68^ included payer perspectives on covering whole-genome versus exome sequencing (tests that provide even greater amounts of genetic variant information compared to MGPTs). Payers described a preference for centers of excellence based on transparency around testing quality and collection of real-world outcome data. By steering patients to such testing centers of excellence, payers could address their concerns that genome sequencing tests are being billed as medically necessary and clinically appropriate. However, payers also recommended that professional societies provide oversight and guidance regarding how these centers of excellence would operate, including specifications for data sharing. The study^69^ provides support for creating closed networks of prequalified labs that agree to perform MGPTs of high quality and participate in public data sharing to improve the clinical interpretation of VUS over time. The same study, however, documented researchers’ and payers’ dissatisfaction with CED, stating that such arrangements are often cumbersome, difficult to implement, and difficult to terminate if results are unsatisfactory. Payers viewed CED as a less attractive policy alternative to improve testing, unless it could be implemented as part of emerging models of value-based contracts.^70^ Both recent interview studies underscore that payers understand the informational complexities of MGPTs and are open to strategies that enable creative approaches to evidence generation and data sharing.

## VI. LIMITATIONS

To date, few payers have emulated the position adopted by Aetna to condition test coverage on data sharing. Potential reasons for payer reticence were elucidated by a modified policy Delphi process focused on identification and evaluation of policy options conducted by the Sulston Project research team.^71^ Participants in the Delphi generated three policy options targeting health insurers: (i) ‘Health insurers could exclude clinical labs from preferred networks if they don’t share’; (ii) ‘Health insurers could penalize reimbursement up to 100 per cent if labs don’t share’; and (iii) ‘Health insurers could reward labs that share data with higher payments.’ In the final Delphi round, the panel of expert stakeholders rated individual policy options on dimensions of effectiveness and feasibility and then selected their top three policy options for each domain (these policy options fell within the ‘incentives’ domain, which contained a total of nine policy options including others targeting research funders, data resources, proficiency testing programs, and journals). Panelists rated the first of the three options (excluding labs that do not comply) more favorably than the other two as both effective and feasible; the second option (penalizing reimbursement for non-compliance) had an overall negative feasibility rating (also, many panelists selected the ‘don’t know’ response for feasibility for the second and third options). That is, the Delphi supported paying labs that agree to share data, which could be accomplished by making data sharing a condition of payment or by including data sharing as a quality metric in payment contracts.

Panelists were prompted to provide the reasoning behind their ratings and rankings. In favor of turning to payers to incentivize data sharing, one noted that ‘a large part of the relevant data will be generated through routine testing by commercial labs’ that are unlikely to share without additional incentives and that payers have the leverage to encourage sharing. On the other hand, some panelists were skeptical that payers would perceive these options as aligned with their interests. For example, one wrote ‘it’s not clear that it’s in their short-term interest to do this’ and that the first option is ‘a draconian step to take for a benefit that’s not health-plan specific’. Another panelist was concerned that making data sharing a checkbox for laboratories could encourage ‘share-washing’, meaning the sharing of unhelpful data for compliance purposes (such as submitting variant classifications to ClinVar without metadata or supporting evidence). One panelist worried about the implications for patients. Related to the first option, they offered the scenario of a patient needing a test from a laboratory that does not share and so having to bear the costs associated with going out of network. Clearly, patients will need to be engaged in how any of these policy options are developed and implemented.

The time may have arrived to build the knowledge commons that can advance the interests of all stakeholders, including payers. The recent announcement from Myriad Genetics to start sharing data with ClinVar in early 2023, reversing its November 2004 policy of not sharing most variant data, is another welcome sign that individual genetic testing laboratories all benefit if they all share data with public databases.^72^ Payers can create incentives to reinforce this trend. As the Trosman study noted,^73^ a significant number of payers were open to supporting use of hereditary cancer panels combining more- and less-studied genes within a model providing rigor and transparency (along the lines of paying for standard care within a research study). But that will also require work by laboratories and professional societies to come to consensus about the implications of a rapidly evolving knowledge base for patient care (eg patient recontact with updated information).

## VII. SUMMARY

While there are strengths and weaknesses to all policy options, the main approaches we propose are (i) to create preferred networks of labs that agree to standard quality practices, including public data sharing; (ii) to make payment conditional on laboratories agreeing to share data sufficient to verify interpretation, including reporting variants and their classification to public databases (ie ClinVar); or (iii) both. Multi-stakeholder engagement (including patients) focused on developing clear steps for implementation, coupled with all parties agreeing on the need and methods for evaluating the effectiveness of these strategies over time, is the most certain path forward.

## Footnotes

1 Mary B. Daly et al., *Genetic/Familial High-Risk Assessment: Breast, Ovarian, and Pancreatic, Version 2.2021, NCCN Clinical Practice Guidelines in Oncology*, 19 J. Natl. Compr. Cancer Netw. JNCCN 77 (2021); Jennifer M. Weiss et al., *NCCN Guidelines® Insights: Genetic/Familial High-Risk Assessment: Colorectal, Version 1.2021: Featured Updates to the NCCN Guidelines*, 19 J. Natl. Compr. Canc. Netw. 1122 (2021).

2 The clinical context for this paper will focus on cancer predisposition testing; however, many of the issues raised are also relevant for tumor profiling as well as other disease predisposition testing.

3 Holly LaDuca et al., *A clinical guide to hereditary cancer panel testing: evaluation of gene-specific cancer associations and sensitivity of genetic testing criteria in a cohort of 165,000 high-risk patients.*, 22 Genet. Med. Off. J. Am. Coll. Med. Genet. 407 (2020); Eric T. Rosenthal et al., *Clinical testing with a panel of 25 genes associated with increased cancer risk results in a significant increase in clinically significant findings across a broad range of cancer histories*, 218–219 Cancer Genet. 58 (2017).

4 Kandamurugu Manickam et al., *Exome Sequencing–Based Screening for BRCA1/2 Expected Pathogenic Variants Among Adult Biobank Participants*, 1 JAMA Netw. Open e182140 (2018).

5 K. Nones et al., *Whole-genome sequencing reveals clinically relevant insights into the aetiology of familial breast cancers*, 30 Ann. Oncol. Off. J. Eur. Soc. Med. Oncol. 1071 (2019).

6 Kathryn A. Phillips et al., *Genetic Test Availability And Spending: Where Are We Now? Where Are We Going?*, 37 Health Aff. Proj. Hope 710 (2018).

7 Janis Geary et al., *Development of an Open Database of Genes Included in Hereditary Cancer Genetic Testing Panels Available From Major Sources in the US*, JAMA Oncol. (2022), https://doi.org/10.1001/jamaoncol.2021.7639 (last visited Mar. 31, 2022).

8 Gene Curation—ClinGen | Clinical Genome Resource, https://clinicalgenome.org/working-groups/gene-curation/ [https://perma.cc/V8ZE-579Q] (last visited July 25, 2022).

9 Daly et al., *supra* note 1; Weiss et al., *supra* note 1.

10 LaDuca et al., *supra* note 3.

11 C. van Marcke et al., *Prevalence of pathogenic variants and variants of unknown significance in patients at high risk of breast cancer: A systematic review and meta-analysis of gene-panel data*, 132 Crit. Rev. Oncol. Hematol. 138 (2018).

12 Wylie Burke et al., *The Challenge of Genetic Variants of Uncertain Clinical Significance*, 175 Ann. Intern. Med. 994 (2022).

13 Melissa J. Landrum et al., *ClinVar: public archive of interpretations of clinically relevant variants*, 44 Nucleic Acids Res. D862 (2016).

14
*Id.*; Heidi L. Rehm et al., *ClinGen — The Clinical Genome Resource*, 372 N. Engl. J. Med. 2235 (2015); Enigma Consortium | Evidence-based Network for the Interpretation of Germline Mutant Alleles, https://enigmaconsortium.org/ [https://perma.cc/H52Y-XZNB] (last visited Aug. 14, 2022); InSiGHT, Lynch Syndrome, https://www.insight-group.org/syndromes/lynch-syndrome/ [https://perma.cc/G7FZ-3GEH] (last visited Aug. 14, 2022).

15 James Casaletto, Melissa Cline & Brian Shirts, *Modeling the impact of data sharing on variant classification*, J. Am. Med. Inform. Assoc. ocac232 (2022).

16 Nandana D. Rao & Brian H. Shirts, *Using species richness calculations to model the global profile of unsampled pathogenic variants: Examples from BRCA1 and BRCA2*, (2022), https://www.medrxiv.org/content/10.1101/2022.11.09.22282146v1 (last visited Jan. 24, 2023).

17 Juli M. Bollinger et al., *BRCA1/2 Variant Data-Sharing Practices*, 47 J. Law. Med. Ethics 88 (2019).

18 Heidi L. Rehm, *A new era in the interpretation of human genomic variation*, 19 Genet. Med. 1092 (2017).

19 Cook-Deegan et al. The Sulston Project: Making the Knowledge Commons for Interpreting Genomic Cancer Variants More Effective. Publication forthcoming, this issue.

20 Rehm, *supra* note 18; Turna Ray, *Genomic variant data sharing gains support; Collaboration seen as key to interpretation challenge – Genomes to People*, genomeweb (2016), https://www.genomes2people.org/wp-content/uploads/2018/08/20160502_GenomeWeb.pdf [https://perma.cc/LC3L-24CH] (last visited July 22, 2022).

21 Aetna Is Helping Lead the Way on the Evolution of Precision Medicine, CVSHealth (2019), https://www.cvshealth.com/newsroom/press-releases/aetna-helping-lead-way-evolution-precision-medicine [https://perma.cc/WWY6-V6BV] (last visited July 22, 2022).

22 Clinical laboratories meeting minimum requirements for data sharing to support quality assurance. ClinGen (accessed 15 June 2023). https://clinicalgenome.org/tools/clinical-lab-data-sharing-list/

23 Cook-Deegan et al., *supra* note 17.

24 Andrew P. Dervan et al., *Payer decision making for next-generation sequencing-based genetic tests: insights from cell-free DNA prenatal screening*, 19 Genet. Med. Off. J. Am. Coll. Med. Genet. 559 (2017).

25 Michael D. Graf et al., *Genetic testing insurance coverage trends: a review of publicly available policies from the largest US payers*, 10 Pers. Med. 235 (2013); Andrew Hresko & Susanne B. Haga, *Insurance Coverage Policies for Personalized Medicine*, 2 J. Pers. Med. 201 (2012).

26 Patricia A. Deverka & Jennifer C. Dreyfus, *Clinical integration of next generation sequencing: coverage and reimbursement challenges*, 42 Suppl 1 J. Law Med. Ethics J. Am. Soc. Law Med. Ethics 22 (2014).

27 US Preventive Services Task Force, *Risk Assessment, Genetic Counseling, and Genetic Testing for BRCA-Related Cancer: US Preventive Services Task Force Recommendation Statement*, 322 JAMA 652 (2019).

28 Genetic Test Assessment, ECRI, https://www.ecri.org/solutions/genetic-test-assessment-payers [https://perma.cc/GJ2Y-278G] (last visited July 22, 2022).

29 Molecular Test Assessment Archives, Hayes, https://www.hayesinc.com/category/publications/precision-medicine/molecular-test-assessment/ [https://perma.cc/S52V-JGHV] (last visited July 22, 2022).

30 Kathryn A. Phillips & Patricia A. Deverka, *The Emerging Use By Commercial Payers Of Third-Party Lab Benefit Managers For Genetic Testing | Health Affairs Forefront*, Health Affairs Forefront (2019), https://www.healthaffairs.org/do/10.1377/forefront.20191021.563154/full/ [https://perma.cc/9MQG-U37N] (last visited July 26, 2022).

31 Geary et al., *supra* note 7.

32 Cook-Deegan et al, *supra* note 17. See also Janis Geary, et al., ‘Equity and Inclusion in Assessing Hereditary Cancer Risk: Insights from Excluded Communities, Structured Interviews, and Population Genetics’ forthcoming in this special issue.

33 CPT® is a registered trademark of the American Medical Association.

34 Centers for Medicare and Medicaid Services, *Billing and Coding: Molecular Pathology and Genetic Testing (A58918)*, MCD Medicare Coverage Database (2022), https://www.cms.gov/medicare-coverage-database/view/article.aspx?articleid=58918&ver=20&= [https://perma.cc/AV48-MFMJ] (last visited July 22, 2022).

35 Robert Cook-Deegan et al., *Impact of Gene Patents and Licensing Practices on Access to Genetic Testing for Inherited Susceptibility to Cancer: Comparing Breast and Ovarian Cancers to Colon Cancers*, 12 Genet. Med. Off. J. Am. Coll. Med. Genet. S15 (2010).

36 Centers for Medicare and Medicaid Services, *supra* note 32.

37 MolDX - Frequently Asked Questions, Palmetto GBA (2022), https://www.palmettogba.com/palmetto/moldxv2.nsf/DID/9A7MFG4181 [https://perma.cc/G3Y6-KGJF] (last visited July 26, 2022).

38 Centers for Medicare and Medicaid Services, *22CLABQ1*, https://www.cms.gov/medicaremedicare-fee-service-paymentclinicallabfeeschedclinical-laboratory-fee-schedule-files/22clabq1 [https://perma.cc/AP4A-A368] (last visited July 7, 2022).

39 CMS Clinical Laboratory Fee Schedule (CLFS) Annual Public Meeting | CMS, https://www.cms.gov/Medicare/Medicare-Fee-for-Service-Payment/ClinicalLabFeeSched/Laboratory_Public_Meetings [https://perma.cc/DWF5-8M7H] (last visited July 22, 2022).

40 Kathryn A Phillips et al., *Payer coverage policies for multi-gene tests*, 35 Nat. Biotechnol. 614 (2017).

41 CMS Clinical Laboratory Fee Schedule (CLFS) Annual Public Meeting | CMS, *supra* note 37.

42 Advisory Panel on Clinical Diagnostic Laboratory Tests | CMS, https://www.cms.gov/Regulations-and-Guidance/Guidance/FACA/AdvisoryPanelonClinicalDiagnosticLaboratoryTests [https://perma.cc/5DQ9-WSTM] (last visited Oct. 26, 2022).

43 PAMA Regulations | CMS, https://www.cms.gov/Medicare/Medicare-Fee-for-Service-Payment/ClinicalLabFeeSched/PAMA-Regulations [https://perma.cc/MRD3-HHQ5] (last visited July 22, 2022).

44 Centers for Medicare and Medicaid Services, *Clinical Laboratory Fee Schedule | CMS*, https://www.cms.gov/Medicare/Medicare-Fee-for-Service-Payment/ClinicalLabFeeSched [https://perma.cc/B7A5-TXLN] (last visited Aug. 14, 2022).

45 Centers for Medicare and Medicaid Services, *Summary of Private Payor Rate-Based Medicare Clinical Laboratory Fee Schedule*, 10 (2022).

46 Janis Geary et al., *Supplemental Files for Payer Incentives for Improving Interpretation of Germline Genetic Testing* (2022), https://osf.io/5fv8w/ (last visited Nov. 2, 2022).

47 Part B National Summary Data File (Previously known as BESS) | CMS, https://www.cms.gov/Research-Statistics-Data-and-Systems/Downloadable-Public-Use-Files/Part-B-National-Summary-Data-File/Overview [https://perma.cc/N6YU-V7KR] (last visited July 7, 2022).

48 Centers for Medicare and Medicaid Services, *supra* note 36 at 1.

49 Geary et al., *supra* note 7.

50
*Id.*

51 Centers for Medicare and Medicaid Services, *supra* note 36.

52
Cigna, *Cigna is clarifying its position on when multi-gene panel tests will be covered* (2018), https://static.cigna.com/assets/chcp/pdf/coveragePolicies/Multi-gene_Panel_Testing_April_2018.pdf [https://perma.cc/EJ9K-PAPB] (accessed 15 June 2023).

53 Edward D. Esplin et al., *Universal Germline Genetic Testing for Hereditary Cancer Syndromes in Patients With Solid Tumor Cancer*, JCO Precis. Oncol. e2100516 (2022).

54 Rebecca K. Marcus et al., *Challenges to clinical utilization of hereditary cancer gene panel testing: perspectives from the front lines.*, 14 Fam. Cancer 641 (2015); Esplin et al., *supra* note 54.

55
UnitedHealthcare West, *Genetic Testing for Hereditary Cancer* (2022), https://www.uhcprovider.com/content/dam/provider/docs/public/policies/signaturevalue-mmg/genetic-testing-hboc-sv.pdf [https://perma.cc/E3Z2-VSM9].

56 MolDX: BRCA1 and BRCA2 Genetic Testing (L36161), https://www.cms.gov/medicare-coverage-database/view/lcd.aspx?lcdid=36161&ver=37&keyword=BRCA&keywordType=starts&areaId=all&docType=NCA,CAL,NCD,MEDCAC,TA,MCD,6,3,5,1,F,P&contractOption = all&sortBy = relevance&bc = 1 [https://perma.cc/H8NV-JMNP] (last visited Jul. 22, 2022); Charles Muller et al., *Underdiagnosis of Hereditary Colorectal Cancers Among Medicare Patients: Genetic Testing Criteria for Lynch Syndrome Miss the Mark*, JCO Precis. Oncol. 1103 (2021).

57 LCD - MolDX: Lab-Developed Tests for Inherited Cancer Syndromes in Patients with Cancer (L38972), https://www.cms.gov/medicare-coverage-database/view/lcd.aspx?lcdid=38972&ver= [https://perma.cc/4GAG-U5AQ] (last visited Oct. 25, 2022).

58 National Research Council (US) Committee on A Framework for Developing a New Taxonomy of Disease, Toward Precision Medicine: Building a Knowledge Network for Biomedical Research and a New Taxonomy of Disease (2011), http://www.ncbi.nlm.nih.gov/books/NBK91503/ (last visited Dec. 26, 2020).

59 Sharing Health Data: The Why, the Will, and the Way Forward, 91 (2021), https://nam.edu/wp-content/uploads/2022/01/Sharing-Health-Data-prepub-FINAL.pdf (accessed 15 June 2023). [https://perma.cc/9NPQ-LQHK] (accessed 15 June 2023)

60 ACMG Board of Directors. Laboratory and clinical genomic data sharing is crucial to improving genetic health care: a position statement of the American College of Medical Genetics and Genomics. Genet Med 2017;19(7):721–22.

61 National Society of Genetic Counselors. Clinical Data Sharing. https://www.nsgc.org/Policy-Research-and-Publications/Position-Statements/Position-Statements/Post/clinical-data-sharing (accessed 15 June 2023)

62 ACMG Board of Directors, *Laboratory and clinical genomic data sharing is crucial to improving genetic health care: a position statement of the American College of Medical Genetics and Genomics*, 19 Genet. Med. Off. J. Am. Coll. Med. Genet. 721 (2017).

63 For example: BlueCross MN, *Preferred Genetic Lab Program*, (2022), https://www.bluecrossmn.com/providers/provider-priorities/preferred-genetic-lab-program [https://perma.cc/R8SC-BEJ6] (last visited Aug. 15, 2022).

64 LCD - MolDX: Lab-Developed Tests for Inherited Cancer Syndromes in Patients with Cancer (L38972), *supra* note 58.

65 Coverage with Evidence Development | CMS, https://www.cms.gov/medicare/coverage/coverage-with-evidence-development [https://perma.cc/L7MG-5KEA] (last visited July 22, 2022).

66 Julia R. Trosman et al., *Payer Coverage for Hereditary Cancer Panels: Barriers, Opportunities, and Implications for the Precision Medicine Initiative*, 15 J. Natl. Compr. Canc. Netw. 219 (2017).

67
*Id.*

68 Kathryn A. Phillips et al., *US private payers’ perspectives on insurance coverage for genome sequencing versus exome sequencing: A study by the Clinical Sequencing Evidence-Generating Research Consortium (CSER)*, 24 Genet. Med. Off. J. Am. Coll. Med. Genet. 238 (2022).

69
*Id.*

70
*Id.*

71 Robinson et al., Policy options to facilitate cancer genomic variant data sharing: Outcomes of a modified policy Delphi. To be published this issue.

72 Myriad Genetics to Submit Hereditary Cancer Risk Variants to ClinVar in 2023, GenomeWeb (2022), https://www.genomeweb.com/molecular-diagnostics/myriad-genetics-submit-hereditary-cancer-risk-variants-clinvar-2023[https://perma.cc/8ZLF-XVR8] (last visited Jan. 24, 2023); Ellen Matloff, *Myriad Genetics (MYGN) To Share Guarded BRCA Variant Information In Public Database*, Forbes, https://www.forbes.com/sites/ellenmatloff/2022/11/03/myriad-genetics-mygn-to-share-guarded-brca-variant-information-in-public-database/[https://perma.cc/J24S-4J53] (last visited Jan. 24, 2023).

73 Trosman et al., *supra* note 65.

74 Source: Policyreporter, quarterly filing of health plans. https://www.policyreporter.com/payer-data/ (accessed 15 June 2023).

